# The Cybathlon promotes the development of assistive technology for people with physical disabilities

**DOI:** 10.1186/s12984-016-0157-2

**Published:** 2016-05-31

**Authors:** Robert Riener

**Affiliations:** Sensory-Motor Systems Lab, ETH Zurich, Tannenstrasse 1, 8092 Zurich, Switzerland; Spinal Cord Injury Center, University Hospital Bagrist, University of Zurich, Zurich, Switzerland

**Keywords:** Competition, Championship, Prostheses, Exoskeletons, Functional electrical stimulation, Wheelchairs, Brain computer interfaces

## Abstract

**Background:**

The Cybathlon is a new kind of championship, where people with physical disabilities compete against each other at tasks of daily life, with the aid of advanced assistive devices including robotic technologies. The first championship will take place at the Swiss Arena Kloten, Zurich, on 8 October 2016.

**The idea:**

Six disciplines are part of the competition comprising races with powered leg prostheses, powered arm prostheses, functional electrical stimulation driven bikes, powered wheelchairs, powered exoskeletons and brain-computer interfaces. This commentary describes the six disciplines and explains the current technological deficiencies that have to be addressed by the competing teams. These deficiencies at present often lead to disappointment or even rejection of some of the related technologies in daily applications.

**Conclusion:**

The Cybathlon aims to promote the development of useful technologies that facilitate the lives of people with disabilities. In the long run, the developed devices should become affordable and functional for all relevant activities in daily life.

## Background

Millions of people worldwide rely on orthotic, prosthetic, wheelchairs and other assistive devices to improve their qualities of life. In the US there live more than 1.6 million people with limb amputations [1] and the World Health Organization estimates the number of wheelchair users to about 65 million people worldwide [2]. Unfortunately, current assistive technology does not address their needs in an ideal fashion. For instance, wheelchairs cannot climb stairs, arm prostheses do not enable versatile hand functions, and power supplies of many orthotic and prosthetic devices are limited. There is a need to further push the development of assistive devices by pooling the efforts of engineers and clinicians to develop improved technologies, together with the feedback and experiences of the users of the technologies.

The Cybathlon is a new kind of championship with the aim of promoting the development of useful technologies. In contrast with the Paralympics, where parathletes aim to achieve maximum performance, at the Cybathlon, people with physical disabilities compete against each other at tasks of daily life, with the aid of advanced assistive devices including robotic technologies. Most current assistive devices lack satisfactory function; people with disabilities are often disappointed, and thus do not use and accept the technology. Rejection can be due to a lack of communication between developers, people with disabilities, therapists and clinicians, which leads to a disregard of user needs and requirements. Other reasons could be that the health status, level of lesion or financial situation of the potential user are so severe that she or he is unable to use the available technologies. Furthermore, barriers in public environments make the use of assistive technologies often very cumbersome or even impossible.

**Fig. 1:**
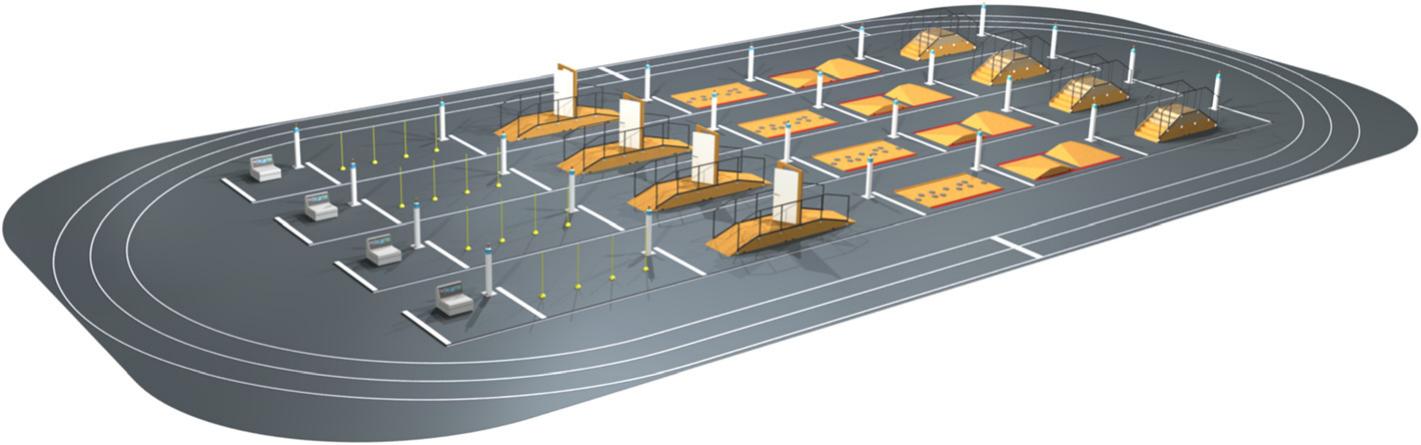
Arena with four parallel race tracks designed for the exoskeleton competition. The pilots start at the left and have to overcome six obstacles with increasing difficulty level

Six disciplines are part of the competition, addressing people with either limb paralysis or limb amputations. The six disciplines comprise races with powered leg prostheses, powered arm prostheses, functional electrical stimulation (FES) driven bikes, powered wheelchairs and powered exoskeletons (Fig. 1). The sixth discipline is a racing game with virtual avatars that are controlled by brain-computer interfaces (BCI). The functional and assistive devices used can be prototypes developed by research labs or companies, or commercially available products. The competitors are called pilots, as they have to control a device that enhances their mobility. The teams each consist of a pilot together with scientists and technology providers, making the Cybathlon also a competition between companies and research laboratories. As a result there are two awards for each winning team in each discipline: a medal for the person who is controlling the device and a cup for the provider of the device (i.e. the company or the lab).

A rehearsal was organized in July 2015 to test race tracks, scoring system and logistics and to generate footage for advertisement. The premiere will take place at the Swiss Arena, in Kloten, Zurich, on 8 October 2016. More information can be found on the Cybathlon website www.cybathlon.com.

## The six disciplines and the challenges in their fields

Pilots with above knee amputations will use actuated prosthetic devices and compete along an obstacle course containing ramps, stairs, doors, soft-cushioned seats, barriers etc. Most of the commercially available leg prosthesis technologies are passive ballistic devices, which are easy to control but make uphill walking and stair ascent challenging. In persons with intact legs, especially the knee joint requires much higher capability of joint power generation during ascent than during level walking [3]. Consequently, users of passive prostheses, including microprocessor-controlled dissipative knee prostheses, have to use hand rails and/or perform an asymmetric non-physiological gait to compensate for the missing power generation in the knee (see for example [4] among many other studies). Powered leg prostheses can induce the missing power and in this way solve these deficiencies; however, the control of such devices is not trivial when interfacing them with the user’s motion intention [5]. Additionally, state-of-the-art batteries are either too heavy or lack sufficient capacity to provide power throughout an entire day. The teams at the Cybathlon will showcase new technologies that might overcome current deficiencies.

**Fig. 2:**
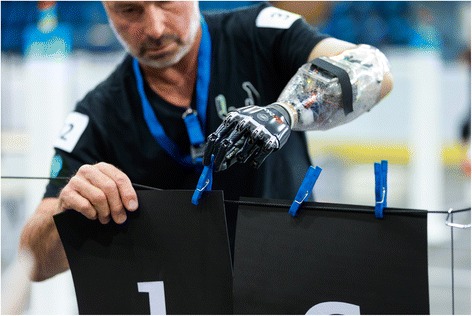
Pilot with a powered arm prosthesis performing a daily living task. Picture was taken at the Cybathlon rehearsal in July 2015 by Alessandro Della Bella, ETH Zurich. The pilot on this image as consented to the publication of this image

At the Cybathlon, pilots with amputations of the lower arm or above will use actuated prosthetic hands and arms to complete various household and food preparation tasks (Fig. 2). The dexterity and versatility of currently available prosthetic hand devices is usually limited with respect to the number of grasps and tasks that can be successfully performed. Therefore, persons with unilateral amputations use their intact arm to perform most daily tasks. Bimanual tasks, which require a high load transfer (e.g., carrying a heavy box) or particular fine motor skills (e.g., opening a small jar of jam) are challenging, because they cannot be solved with most state-of-the-art upper arm prostheses. Consequently, up to 60 % of people with upper-limb amputation fitted for conventional upper-limb prosthetic device fail to use it regularly or reject it altogether [6, 7]. The high rejection rate of upper limb prostheses has been attributed to poor training, late fitting, limited usefulness especially for the users with more proximal amputations, and various other factors. Significantly lower rates of rejection can be seen for more advanced, i.e. body-powered (26 %) and electric (23 %) devices [8].

Four out of the six disciplines of the Cybathlon address people with limb paralysis of varying degrees after lesions such as spinal cord injury: Pilots with complete paraplegia will compete in a bike race, where FES devices assist them in performing pedaling movements. FES technology has been used for movement restoration for decades, but has not achieved satisfactory performance due to limitations in setup-time, movement controllability, muscle force magnitude, muscle selectivity and fatigue resistance [9, 10]. Most promising stimulation systems are implanted, as they yield better selectivity and higher force output than non-invasive systems [9, 11]. However, there are drawbacks with respect to invasiveness, risk of infections and costs. Because of these deficiencies, current FES technology has not been accepted by physicians and patients for daily clinical routine [12].

In both the powered wheelchair race and the powered exoskeleton race, pilots with paralysis will master obstacle courses with ramps, stairs, bends, doors and uneven terrain (Fig. 1). More and more companies offer advanced and powerful solutions for wheelchairs. However, control technology does not provide adequate mobility and comfort for many electrically powered wheelchair users, especially under adverse driving conditions [13]. Wheelchair accessibility in public buildings is still limited despite the enforcement of existing laws and regulations [14]. Most outdoor devices are too bulky and not agile enough for indoor use, whereas commercial indoor wheelchairs are not capable of overcoming uneven terrain or steps. So called intelligent or smart wheelchairs have been available for decades, but have not yet been adopted by a large portion of the population [15, 16]. An alternative to wheelchairs are exoskeletal devices that assist people with paraplegic lesions during gait in the upright position [17, 18]. However, battery power is limited to a few hours of operation and the devices are still very bulky and heavy. Most of the commercially available multi-joint exoskeletons have weights in the range of 21–28 kg, with the device “REX” reaching a weight of almost 40 kg [17, 19]. Furthermore, current commercial systems have a limited number of degrees of freedom and reduced ranges of movements preventing the devices from gait on inclined surfaces or stairs. Thus, exoskeletal devices are not yet a realistic alternative for lightweight, energy efficient, and often foldable manual wheelchairs.

In the BCI race, pilots with paralysis of all four limbs will control a virtual avatar in a racing game displayed on a computer screen. The best pilots will be able to distinguish three different commands to overcome three different kinds of virtual obstacles and, thus, will be rewarded by a temporal advantage in the game. A wrong command or a command with too long latency will be penalized by decelerating the avatar on its track. BCI technology is becoming more and more popular, however most systems only function accurately in a lab environment [20]. The time needed for device setup, comfort, cosmetic aspects, function and reliability are still not satisfactory and have prevented broad use and acceptance outside labs [21].

## Conclusion

The Cybathlon will provide a platform that encourages exchange between people with disabilities or physical weaknesses, the research and development world, funding agencies, and the general public. In this way, the Cybathlon aims to promote the development of useful technologies that facilitate the daily lives of people with disabilities or physical weaknesses and provide the basis for more independence. In the long run, the developed devices should become affordable and functional for all relevant activities in daily life.

Cybathlon can also be considered as a complement to the Olympic or Paralympic games. In contrast to the Paralympic games, it allows the use of any kind of technical aids, thus also enabling people with more severe disabilities to participate in a competition. The goal is not to be the fastest and the strongest among the participants, rather the goal is to be the most skilled pilot who utilizes advanced technologies in ways that allow the challenges of everyday life to be overcome with ease.

## Abbreviations

BCI, Brain-Computer Interface; FES, Functional Electrical Stimulation; US, United States
